# Agriculture land use transformation: A threat to sustainable food production systems, rural food security, and farmer well-being?

**DOI:** 10.1371/journal.pone.0296332

**Published:** 2024-01-24

**Authors:** Meiyi Li, Junrong Li, Shamsheer ul Haq, Muhammad Nadeem

**Affiliations:** 1 School of Business, Tianshui Normal University, Tianshui, Gansu, China; 2 School of Public Administration, University of Electronic Science and Technology of China, Chengdu, Sichuan, China; 3 Department of Economics, Division of Management and Administrative Science, University of Education, Lahore, Pakistan; 4 College of Economics and Management, Nanjing University of Aeronautics and Astronautics, Nanjing, PR China; 5 Department of Economics, Virtual University, Islamabad, Pakistan; University of Sargodha, PAKISTAN

## Abstract

The acquisition of agricultural land is a crucial aspect of survival for numerous rural communities, serving as a fundamental tool for combating poverty and food insecurity and promoting equitable sustainable economic progress. The expropriation of land offers a promising prospect for remedying past inequities and promoting both economic progress and food sufficiency. Limited research has examined the association between land expropriation and food security, livelihood shocks, and the well-being of rural households worldwide. Therefore, this research explores the implications of land expropriation on food security, livelihood shocks, and well-being of land lost rural communities. The data were collected from 384 farmers selected through stratified sampling techniques using face-to-face surveys in rural China. The data were analyzed using descriptive and logit regression models. The descriptive findings showed that land expropriation has detrimental effects on the livelihood, food security, and well-being of the farmers. Furthermore, these impacts are more harmful among land-expropriated households with a lower educational level, a large family size, and women farmers in less developed rural communities. The econometric results evinced that gender, age, education level, marital status, family size, and negative changes in income all significantly affect the impact of land expropriation on the food security of farmers. Similarly, the findings revealed that farmers with lower education levels were more likely to be affected by land loss as compared to farmers with medium and high education levels. Farmers with complete land loss were 1.70 times more likely to suffer livelihood shocks than those with partial land loss. The results also evinced that the well-being of all farmers was not affected equally, and some farmers’ well-being was affected more than others due to various socioeconomic backgrounds. Therefore, this study suggests the implementation of public policies that provide support to farmers who have been marginalized due to land acquisition.

## 1. Introduction

The process of industrial development and growth in urbanization has led to the transformation of farming land to non-farming uses. This phenomenon has been observed in industrialized nations and is currently taking place in emerging economies especially those experiencing rapid economic transition, such as China [1]. Following China’s economic openness and reform in 1978, there has been a remarkable surge in urbanization in the country, encompassing both the magnitude of its urban population and the extent of land that has been transformed into urban areas [2,3]. The extensive utilization of rural land for projects to promote development has resulted in the displacement of numerous farmers from their land and relocation of farming communities. It occurred concurrently with the rapid expansion of urban areas [4,5]. Zhong et al. [6] asserted that China is a prominent nation globally, characterized by extensive land utilization, significant transformations, and severe land disputes. The urban development land area has experienced a significant increase from 6. 72 thousand km2 to 49.983 thousand km2, resulting in a net growth of 43.26 thousand km2, which represents a 6.44-fold increase. Similarly, the mean yearly expansion rate reaches a maximum of 6.27% [7].

The primary asset of utmost significance for the vast majority of individuals residing in rural areas is land [8]. This is due to its dual functionality as an on-going source of livelihood and as a means of social insurance. The rapid growth of industrial development and urbanization in China since the 1990s has resulted in the expropriation of a substantial portion of rural collective land. The confiscation of arable land totaled 1600 thousand hectares between 1987 and 2000. Subsequently, due to the accelerated pace of urbanization post-2006, the mean expropriated land area has surged to 0.4 million hectares annually [9]. The phenomenon of land expropriation has resulted in a significant number of farmers experiencing land loss simultaneously. More than 500 million farmers in China have lost their land completely or partially due to expropriation [10,11]. It is projected that the expropriation of farming land by the government will amount to 2530 thousand hectares till 2030. This is expected to result in the displacement of over 80 million farmers who have lost their land [12]. The phenomenon of land expropriation has resulted in a significant number of farmers experiencing land loss simultaneously. Expropriation has resulted in the complete or partial loss of farming land owned by around 500 million farmers in China. It is projected that the expropriation of farming land by the government will amount to 2530 thousand hectares in the country. This is expected to result in the displacement of over 80 million farmers who have lost their land [12]. The issue at hand would be of greater magnitude in the peri-urban regions surrounding major and intermediate cities, as well as in the advanced localities that are marked by a substantial populace and limited land resources. The issue at hand would be of greater magnitude in the peri-urban regions surrounding major and intermediate cities, as well as in the advanced localities that are marked by a substantial populace and limited land resources [13].

The issue of land expropriation in China has resulted in the formation of land-lost farming communities and has given rise to various social and economic challenges. These include concerns regarding the equitable distribution of land compensation, disputes arising from the expropriation of agricultural land, and the sustainability of the livelihoods of land-lost farmers [14–17]. The phenomenon of opposition to land expropriation in China has resulted in individuals being labeled as ’Three-No wanderer”, indicating having no land, no employment, and no basic welfare assurances. This situation has arisen despite attempts to provide compensation and improve land governance [18]. The expropriation of land has been identified as the primary cause of disturbances in peri-urban and rural regions of China [17,19]. The matter of land dispossession is a matter of concern due to its major consequences for reducing poverty and development of the economy particularly with regards to access to land and the protection of property [20]. Land offers not only a means of securing a livelihood and generating direct economic gains, but also holds advantages such as inheritance and the possibility of being sold or mortgaged during times of financial need [21].

The act of forced land dispossession presents substantial risks to the welfare of farmers. The proportion of farmers who have lost their land have transformed into migrant workers, looking for non-agricultural employment opportunities or establishing their own entrepreneurial ventures [22]. However, a significant number of these farmers are left unemployed. Some individuals engage in temporary and part-time employment that is characterized by meager remuneration and challenging working conditions [23]. The challenge of restoring livelihoods often results in a decrease in income for the majority of farmers who have lost their land, which is a prominent negative consequence of dislocation [24]. In addition, it should be noted that although farmers who have lost their land may have been incorporated into the household registration system as urban residents, they are not entitled to the same extent of social welfare benefits as those who are officially recognized as urban citizens [4].

Similarly, the main effects of domestic land expropriation in the nation include urban spread out, land being turned into a commodity for food security, and agricultural modernization. Consequently, individuals without land tenure are encountering the potentiality of joblessness, difficulties in achieving food security, and inadequate handling of relocation efforts. Furthermore, it has been observed that the per-capita cultivated land in China is significantly lower than the global average [25]. This situation poses a significant challenge in terms of ensuring food security for the growing urban population [26]. Research conducted on a group of landless peasants has revealed that the hukou registration system has resulted in a lack of social security benefits for the majority of them following land dispossession. Additionally, a minority of this group experienced unemployment after displacement [15,27]. Under such conditions, individuals who migrate from rural areas are susceptible to unemployment and food insecurity [28].

The on-going urbanization in China has brought to light a multitude of significant issues, including but not limited to the exacerbation of the urban-rural divide, environmental degradation, rising populations, and resource scarcity. Huang et al. [29] identified land issues as the primary challenges that impede the stability and sustainable development of rural areas in China. The expropriation of land in China has resulted in a variety of contradictions. The alteration in land use policy results in an increase in political and social instability, discrimination, and food insecurity [24]. The aforementioned circumstances have led to an increase in land-related disputes due to the continued shortage of land, coupled with a surge in growing populations and urban expansion. The agricultural workers, who are concerned about the potential loss of their primary means of production without appropriate recompense, engaged in a sequence of coordinated acts of forceful opposition against the government and its representatives. Although Chinese peasants heavily depend on farmland for their livelihood, the government initiated economic development plans that necessitated the allocation of vast areas of land for the establishment of factories, marketplaces, and infrastructure development [30]. The imperative to acquire land for said objectives places significant strain on the Chinese government, necessitating the initiation of farmland use policy for negating the implications of expropriation on rural community. Thus, the continuous expropriation of farm land in China affects rural residents adversely. Keep in mind the issues created by land expropriation in rural areas; this study explores the consequences of farming land acquisition on the food security, livelihood shocks, and well-being of land lost farmers.

The main cause of food insecurity is low agricultural productivity, which is related to declining land ownership per person, population growth, climate change, and poverty [31]. Several recent pieces of data from China and other countries indicate that the focus of land expropriation has shifted toward conveniently available productive land [32]. There is disagreement on how current land acquisition will affect food production in rural communities. Proponents of land acquisition stress that these land acquisitions improve rural communities’ access to and availability of food. However, detractors have highlighted that land acquisition negatively affects food security in rural communities [33]. This is because they limit access to water resources and productive agricultural land, which also affects their livelihoods [32]. Researchers are divided according to the degree to which land acquisition contributes to food security. Moreover, earlier studies on the effects of land acquisition have focused more on the general socioeconomic effects felt by larger groups than on the implications for the livelihood, food security, and general well-being of farmers. Therefore, this research explores the implications of land expropriation on food security, livelihood shocks, and well-being of land lost rural communities [34].

This study has the potential to benefit various stakeholders. First, policymakers have the opportunity to employ these findings to develop sustainable land use plans. This will effectively reconcile agricultural expansion with environmental preservation, guaranteeing enduring food security. Agricultural professionals have the potential to acquire valuable knowledge regarding resilient farming techniques that can effectively address the adverse consequences of land use alterations. This knowledge can contribute to enhancing agricultural productivity, while simultaneously preserving ecological well-being. Moreover, it is worth considering that rural communities might potentially experience positive outcomes through the implementation of specific interventions aimed at bolstering food security and enhancing the overall welfare of farmers. These interventions would primarily focus on resolving the various obstacles and difficulties involved in the shift in land use. This research could potentially provide significant value to scholars and experts in the field, as it contributes to the expanding pool of information on the complex interplay between land-use patterns and the achievement of sustainable food production. In general, our research holds promise for informing comprehensive strategies aimed at protecting food systems, rural livelihoods, and the welfare of farmers amidst changing land use dynamics.

## 2. Review of literature and hypothesis development

The process of rapid urbanization necessitates the phenomenon of land expropriation [4,16,35]. Throughout history, the government has commonly used land expropriation as a means to fulfill the needs of public infrastructure construction or urban development. Following China’s reform and opening up in 1978, there has been a significant surge in industrialization and urbanization, leading to the expropriation of a substantial portion of farmland belonging to farmers for the purpose of public infrastructure and urban industrial and commercial development. Based on survey data provided by the National Development and Reform Commission, it has been observed that during the period spanning from 1978 to 2003, over 70% of the farmland that was utilized for non-agricultural construction purposes in China was procured through expropriation. China’s urbanization development has accelerated significantly since 2002, leading to a substantial increase in the conversion of farmland to urban areas [36]. The land expropriation involves the conversion of rural land from agricultural usage to urban construction usage. According to previous research [37,38], land represents the fundamental resource for agricultural production, and any expropriation of land can have a significant negative impact on the amount of cultivated land. The process of large-scale land expropriation has significant implications for the daily lives and agricultural output of indigenous farmers. The expropriation of land results in a decline in agricultural production [39]. The agricultural land is a fundamental resource that guarantees the sustenance of food security, as stated by Chaplin-Kramer et al. [40] and Foley et al. [41]. By increasing farming production, it is possible to exaggerate and increase the utilization of farmland, resulting in elevated production levels and improved food security for farm families [42,43]. The expropriation of land has been observed to diminish the availability of farmland for family members. Furthermore, instances of unjust land expropriation have led to the displacement of numerous individuals, thereby contributing to food insecurity, societal breakdown, and a decline in cultural unity [44]. Thus, we hypothesize that

H1: The land expropriation affects the food security of the farmers.

Agriculturalists constitute the primary victims of land expropriation initiatives, and their subsistence is characterized as their means of livelihood centered on their abilities, resources, and undertakings [45–47]. The implementation of land expropriation projects in China has resulted in notable alterations to the means of livelihood for farmers and their families [48–50]; it is a common practice of the Chinese government to offer a guarantee of enhancing the standard of living of farmers prior to implementing any reforms related to rural land tenure. According to Zhou and Fu’s [51], land expropriation resulted in both short-term and long-term alterations to individuals’ means of subsistence. The remuneration provided for land expropriation is a significant determinant of the impact on immediate livelihoods. The sustenance of livelihoods over an extended period of time is intricately connected to both stable employment and the provision of social security [52]. The inadequate compensation standards for land expropriation and the singular mode of resettlement have a significant impact on the farmers’ livelihoods [1]. The farmers’ livelihood resources may be compromised and difficult to sustain due to issues such as an imperfect system of land expropriation, leading to their deterioration. The expropriation of land poses challenges to the livelihoods of farmers, primarily attributable to their limited employment opportunities [53,54], inadequate protection of their rights and benefits, and suboptimal social security provisions [55,56]. The expropriation of land did not result in an increase in wealth for farmers and instead led to a significant number of households being classified as "three-no-farmer households," meaning they had no access to farmland, employment opportunities, or subsistence allowances. Gan and Sun [57] stated that farmers who lost their land due to expropriation commonly encountered unemployment along with little income. This can be attributed to their limited schooling years and absence of exposure to non-farming employment opportunities [58]. The land expropriation resulted in adverse effects such as the deprivation of sustenance, disruption of commercial activities, emotional suffering, and disputes over land among producers whose lands were expropriated. The payment system quality was inadequate and the system for social well-being was imperfect, resulting in a decrease in the standard of living for farm families who lost their land. Farmers were not well-suited to urban living due to unfamiliar rules and regulations and social exclusion [59]. Conflicts related to property rights, resources, and development arose during land expropriation, leading to a reduction in the livelihood and well-being of farmers who lost their land [60]. Thus, we formulized the following two hypotheses:

H2: The land expropriation influences the farmers’ livelihood.
H3: The land expropriation affects the well-being of farmers.

## 3. Materials and methods

### 3.1. Questionnaire design

Questionnaire surveys are commonly employed by researchers to gather primary data and examine the consequences of agricultural land acquisition. The initial step involved conducting a comprehensive review of pertinent scholarly literature, followed by seeking input from academic and research experts with expertise in a specific field of study. This collaborative effort was undertaken to develop a survey instrument for this study. A two-stage technique was used to assess the suitability and reliability of the questionnaire. In the initial phase, a cohort of three scholarly experts, including professors and researchers, undertook a comprehensive examination and evaluation of the questionnaires. These individuals possess high levels of expertise in agricultural economics. The objective of this study was to ascertain the degree to which the questionnaire encompassed all pertinent information and evaluate the comprehensibility of technical terminology. The initial evaluation involved the utilization of a sample group of 15 individuals. Consequently, modifications were implemented, and the questionnaire was modified after its initial completion. The aforementioned modifications were included in the final questionnaire, and a meticulously crafted questionnaire was used to gather data for the study. The rationale behind selecting Chinese as the language for the questionnaire design was primarily influenced by the widespread usage of this language among the target audience. Attempts were made to minimize the potential for the loss of information when translating from Chinese to English. In this way, a well-designed questionnaire prepared for this study before data collection.

The survey instrument was partitioned into multiple pieces. The initial segment included inquiries pertaining to the socioeconomic attributes of farmers who experienced land loss. The second section of the questionnaire assessed the food security status of the farmers using a range of statements. The final phase of this study assessed the impact of land acquisition on livelihood. The fourth section comprises assertions pertaining to the subjective well-being of farmers.

### 3.2. Study area and data collection

This study employs a quantitative approach to investigate the determinants of household food security, livelihood differentiation, and well-being among Chinese farmers who have experienced land expropriation in four most significant economic regions of China (Cheng-Yu region, the Circum-Bohai Sea region, the Yangtze River Delta region, and the Pearl River Delta region). The survey was conducted among villagers in China to gain insights into the implementation of land expropriation at the village level. The focus of this study is on individuals residing in rural areas who have been deprived of their land, commonly referred to as land-dispossessed villagers. In order to optimize the scope of the intended demographic, a purposeful selection of provinces and cities was made to emphasize the matter of land expropriation. Initially, four regions exhibiting accelerated urbanization were identified. In each geographical region, a stratified sampling approach was employed, whereby cities were categorized into three strata based on their population size. One city was then randomly selected from each stratum to be included in the sample. The aforementioned methodology produced a cumulative count of 12 urban centers including Chengdu, Yueqing, Chongqing, Jiangyiin, Zhongshan, Ningbo, Guangzhou, Nanchong, Jinan, Sanhe, Weifang, and Dongguan. Subsequently, five villages were chosen within each of the aforementioned cities, resulting in a total of 60 representative villages for the study. A deliberate sampling strategy was employed to select 20 households from each village. A total of 384 samples were obtained through this process from 15 October to 16 November 2022, indicating incidents of land expropriation.

A team of researchers, consisting of undergraduate as well as graduate students who had undergone prior training, carried out in-person interviews with the heads of selected households at their respective residences. The responses were subsequently collected on a questionnaire in paper format. The survey was conducted with complete anonymity. In instances where a survey participant was unavailable, we conducted additional visits to ensure the completion of the questionnaire.

Although the data were analyzed anonymously, the study was approved by the ethics committee of Nanjing University of Aeronautics and Astronautics, China. Informed verbal consent was obtained from all study participants. For this purpose, a statement about the purpose of the study was written at the beginning of the survey questionnaire and read to the study participants before beginning the survey. In this way, information was gathered from only those participants who gave their consent to participate in this study.

### 3.2. Dependent variable measurement

The subject of interest pertains to the effects of land expropriation on the food security, livelihood shocks, and overall well-being of farmers. The initial factor, denoted as "food security," pertaining to the household was assessed through GFIESS-M [61]. The GFIESS-M is composed of eight binary (yes/no) items. The Food and Agriculture Organization (FAO) has developed the GFIESS-M, a tool that encompasses all four dimensions of food security and is employed to assess the advancement of countries towards SDG-2, which aims to eradicate global hunger. Regarding their experiences with food insecurity both before and after land expropriation, the head of the farm family was questioned. The FAO [62] criteria were utilized to classify households into three distinct categories depending on the raw scores of the farm families. A value of 1 was given to an indicator when it made a favorable contribution to the issue of food insecurity, while a value of 0 was given to it otherwise. The raw scores of the participants exhibited variability ranging from 0 to 8. A household was classified as food insecure if its score was equal to or exceeded one, while a score of 0 indicated food security. Households were classified as moderately or severely food insecure if their raw score was equal to or higher than 4, and as food secure if their raw score was 0. A household’s level of food security was determined based on their raw score, with a score of 7 or 8 indicating severe food insecurity and a score of 0 indicating otherwise. Furthermore, in order to assess the impact of the land expropriation on affected families’ food security, the scores obtained by every family after the land expropriation were compared to their normal period (before land expropriation) scores using the Food Insecurity Experience Scale (FIES). A positive difference (>0) between the two scores indicated a decline in food security for the household. Thus, the difference greater than or equal to 1 was assigned “1”, otherwise "0," to develop the dependent variable. A similar method was employed by Shahbaz et al. [63] to explore the food security affected by COVID-19.

The farmers who suffered livelihood shocks due to land expropriation were assigned a vale of “1” otherwise “0” for further analysis. Similarly, the farmers whose well-being was affected due to land expropriation were assigned a vale of “1” otherwise “0” for using it as a dependent variable in the econometric model.

### 3.3. Econometric models

Due to the binary nature of the dependent variables, including food security, livelihood shocks, and the well-being of farm families, the logit model was applied to explore the impact of land expropriation. Thus, three separate logit models were executed; their general model descriptions are given below in the Table 1.

**Table 1:** Model specifications.

Variables	Model-1	Model-2	Model-3
Dependent	Food security (1,0)	Livelihood differentiation (1,0)	Well-being (1,0)
Model specification	$y_{i1}^{*}=\beta X_{i}^{\prime }+\gamma_{i1}$	$y_{i2}^{*}=\beta X_{i}^{\prime }+\gamma_{i2}$	$y_{i3}^{*}=\beta X_{i}^{\prime }+\gamma_{i3}$

The variable $y_{i}^{*}$ represents a binary variable that is not directly observable, and the subscript i denotes the specific household that has experienced food security as a result of either land expropriation in Model-1, livelihood differentiation in Model-2, or reduced well-being in Model-3. Xi represents a set of socioeconomic attributes of a household that govern the effects of land expropriation on food security, differentiation of livelihoods, and the overall welfare of the impacted families. The vector of regression coefficients is represented by β, while the error term is denoted by γi. The latent variable $y_{i}^{*}$ can not observed directly. It can be observed only

$$y_{i}=\left\{ \begin{array}{c} 1\;if\;y_{i}^{*}>0\\ 0\;if\;y_{i}^{*}\leq 0 \end{array}\right.$$

The variable "*y_i_*" serves as an indicator of the effect of land loss on the food security, livelihood differentiation, and overall well-being of farm families. Specifically, if the expected deterioration is higher ($y_{i}^{*}>0$), it suggests that the aforementioned factors have been negatively affected by land expropriation. Conversely, if the expected decline is less than or equal to 0 (yi * i ≤ 0), it indicates that the land expropriation has not had a significant impact on the food security, livelihood shocks, and well-being of farm families. Therefore, above equation can be interpreted in relation to a binary variable that has been observed.


$$pr(y_{i}=1|X_{i})=\emptyset (\beta X_{i}^{\prime })=\frac{e^{\beta X_{i}^{\prime }}}{1+e^{\beta X_{i}^{\prime }}}=\frac{\exp\left(\beta X_{i}^{\prime }\right)}{1+\exp\left(\beta X_{i}^{\prime }\right)}$$

The function ∅ (.) is applied to a particular binomial distribution, as described by Fernihough [64].

The coefficients in isolation do not provide a comprehensive understanding of the extent to which changes in the socioeconomic characteristics of households will impact the probability of the effects of land loss on food security, livelihood differentiation, and the well-being of affected farmers. In order to provide a quantitative and comprehensive analysis, we computed the odds ratios for the explanatory variables.

## 4. Results and discussion

### 4.1. Socioeconomic background of farmers

Socioeconomic characteristics reveal important information about the background of the farmers [65]. The gender of the farmers is an important socioeconomic characteristic and might be a crucial factor in determining the influence of farming land acquisition on the socioeconomic well-being of the farming community. More than half of the farmers participating in this study were male. The age of the farmers is also an important determinant, and farmers were categorized into young and old categories. Farmers with ages less than 40 were categorized as young farmers, and those with ages greater than 40 were categorized as old farmers. The descriptive results revealed that more than half of the farmers in this study were young farmers. Similarly, farmers were divided into three categories with respect to their education level. Farmers with primary schooling were included in the low-level education group. Framers with middle and high school education were part of the medium-level education group, and all others were included in the high-level education group. More than one-fifth and two-fifths of farmers had a low and high level of education, respectively. A large majority of the farmers participating in this survey were married, and the majority of the participants had small families (<4 members).

Land is mainly expropriated for three main purposes: i) the development of economic zones; ii) infrastructure expansion; and iii) real estate. Therefore, the land expropriation purpose variable was divided into these three categories. Half of the farmers reported that their land had been taken by real estate developers. The development of economic zones was the second most reported cause behind land expropriation in this study. In recent times, China has shifted its policy from job compensation to monetary compensation. Therefore, a large majority of the farmers received monetary compensation for their expropriated land. More than three-fifths of the farmers reported a negative change after land expropriation. More than two-thirds of the farmers reported that their land was completely expropriated. The average distance of rural households was more than fifteen kilometers from the city centers (Table 2).

**Table 2:** Socioeconomic background of farmers.

Farmer characteristics	Mean (Std. dev.)
Gender (1 = Male, 0 = Female)	0.57 (0.47)
Age (1 = Young, 0 = Old)	0.54 (0.44)
Education level	
Low	0.21 (0.41)
Medium	0.36 (0.45)
High	0.43 (0.44)
Marital status (1 = Married, 0 = Single)	0.76 (0.34)
Family size (1 = Small, 0 = Large)	0.67 (0.38)
Land expropriation purpose	
Development of economic zone	0.29 (0.54)
Infrastructure expansion	0.21 (0.37)
Real estate	0.50 (0.49)
Land expropriation compensation (1 = Monetary, 0 = Non-monetary)	0.90 (0.27)
Change in household income (1 = Decreased, 0 = Others)	0.61 (0.40)
Expropriated land (1 = Fully, 0 = Partially)	0.67 (0.39)
Distance to city centre (Km.)	15.76 (7.54)
Distance to city centre (Km.)	15.76 (7.54)

### 4.2. Consequences of farming land loss on food security of farming community

**Fig 1:**
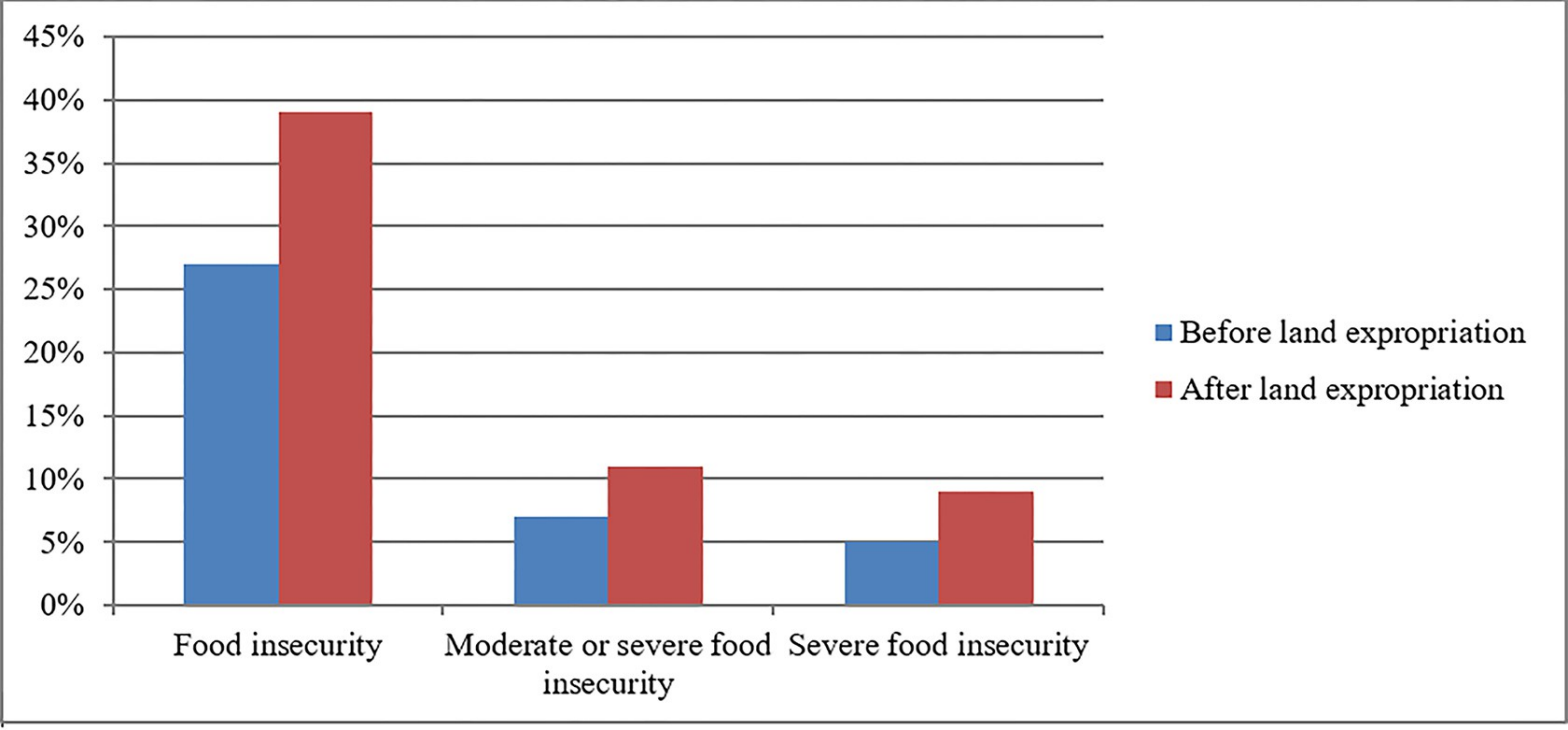
Consequences of farming land loss on food security of farming community.

Fig 1 shows the consequences of land loss on the food security of the farming community. The results indicated that food insecurity increased significantly after land expropriation as compared to before land expropriation. Rural areas in developing countries are home to poverty and food insecurity, and China is no exception. The results revealed that 27% of households were food insecure before land expropriation, which increased to 39% after land expropriation. Similarly, moderate or severe food insecurity also increased by 4% after land expropriation in rural areas of China. Moreover, severe food insecurity among farming households also increased significantly after land expropriation compared to before land expropriation in rural areas. The implementation of unjust land acquisition practices has led to the displacement of numerous individuals, thereby contributing to food insecurity in rural areas [44]. Mabe et al. [66] stated that land expropriation has a significant impact on households’ food security, and livelihood sustainability. They also reported that the food security of farmers without land expropriation was better than that of farmers with land expropriation. The results corroborate Cotula’s [67] findings that rural families’ food insecurity worsened as a result of land expropriation. Randolph [64] also found that land expropriation increases food insecurity among farming community.

### 4.3. Factors determining the effect of land loss on food security

The logistic regression results showed that various factors affect the effect of farming land loss on food security (Table 3). Male farmers were 0.39 times less likely to face declined food insecurity due to land expropriation as compared to female farmers. Similarly, young farmers were 0.84 times less likely to be face worsened food security after land expropriation than old farmers. Many issues, such as that concerning policy execution, remain unresolved, in addition to the inadequate payment threshold [68,69]. The comparatively small compensatory amount cannot compensate for the loss suffered by landless farmers, resulting in deterioration in their living conditions and food insecurity [70]. The farmers with low and medium level of education had respectively 1.05- and 1.02-times higher probability of suffering food insecurity due to land expropriation than those with high level of education. The results also indicated that married farmers’ food security is 1.07 times more likely to be affected due to land expropriation.

**Table 3:** Factors determining the influence of land loss on food security.

Variables	β	Std. errs.	Odd ratios
Gender (1 = Male)	-0.941***	0.530	0.390
Age (1 = Young)	-0.177***	0.091	0.84
Education level (a)			
Low	0.056**	0.026	1.05
Medium	0.026**	0.016	1.02
Marital status (1 = Married)	0.073*	0.020	1.07
Family size (1 = Small)	-0.008*	0.001	0.99
Land expropriation purpose (b)			
Development of economic zones	-0.076	0.065	0.92
Infrastructure expansion	-0.002	0.003	0.99
Land expropriation compensation (1 = Monetary)	-0.082	0.064	0.91
Change in household income (1 = Decreased)	0.118*	0.021	1.13
Expropriated land (1 = Fully)	0.472*	0.125	1.61
Distance to city market (Kilometres)	0.077	0.071	1.08
LR chi2	279.33		
Prob. > chi2	0.000		
Log likelihood	1476.54		

(a) = base category high education level, (b) = base category real estate.

The food security of farmers with small family sizes is 0.99 times less likely to be affected by land loss compared to farmers with large family sizes. The reason may be that agriculture plays a vital role in food security, especially in rural areas [71,72]. Self-production is the key to ensuring food security in rural areas. After land loss, it might be easy to fulfil the nutritional demands of small families through non-agricultural sources. Farmers reporting a decrease in their income after expropriation were 1.13 times more likely to suffer deteriorated food security than those who did not experience a decrease in their income. Many farmers, after land loss, are forced into poverty because they lack a safe place to live [73]. They have challenging financial and living situations as they try to fit into urban life while attending to various requirements [74]. Farmers with full land losses were 1.61 times more likely to suffer food insecurity than those whose land was partially expropriated. Meanwhile, the social security system is subject to significant criticism. According to Guo [75], the social security policy for those without land, which encompasses pension, medical, and employment insurances, is still inadequate. The living stipends obtained by landless farmers were sourced from the revenue generated subsequent to the transfer of land rather than being provided by governmental bodies, village collectives, or individual farmers. This further aggravates the food and nutrition security of the farmers after land expropriation.

### 4.4. Effects of land expropriation on livelihood shocks

**Fig 2:**
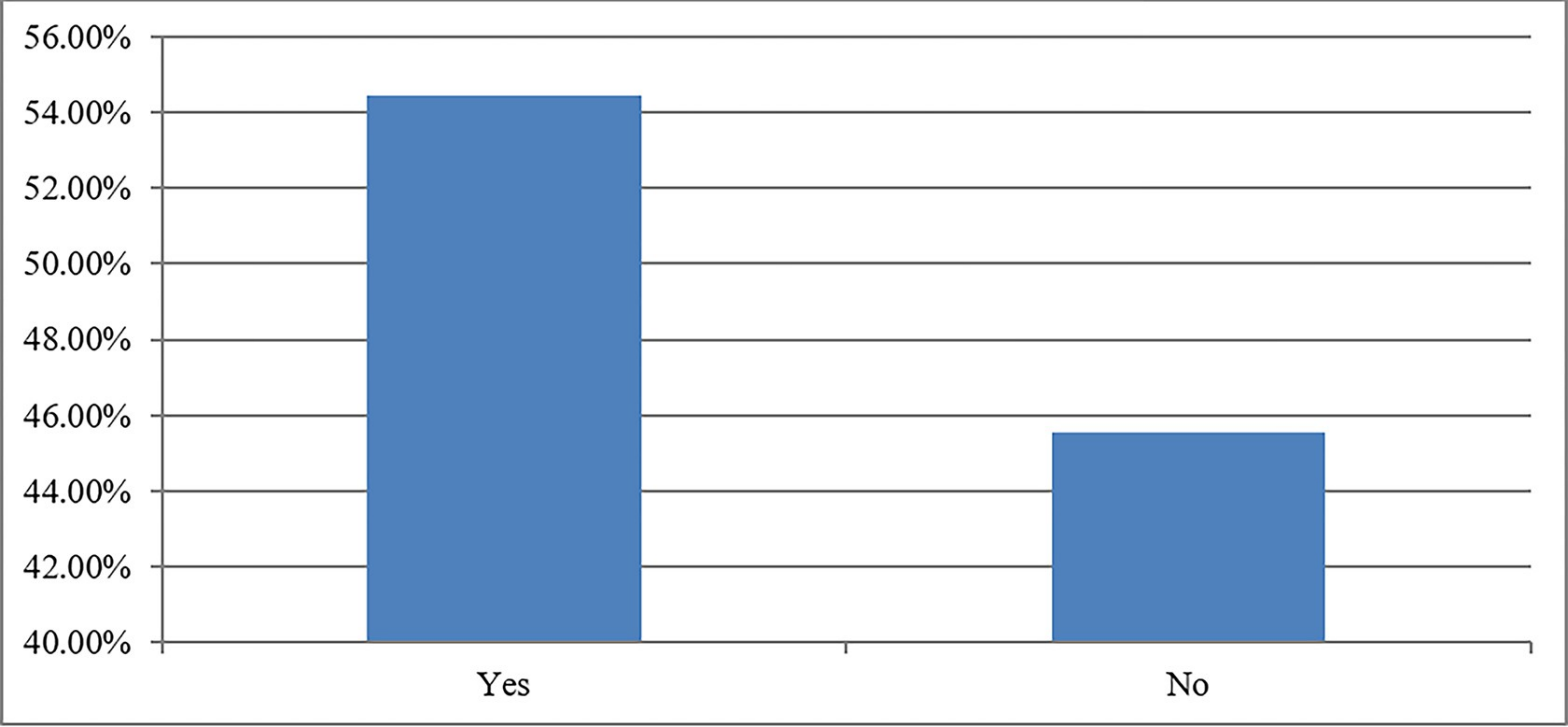
Farmers who suffered livelihood shocks due to land expropriation.

The non-agricultural use of farming lands is increasing alarmingly, posing a threat to the livelihood of farming communities and agricultural sustainability in developing countries. Expropriated lands contribute a large share of the revenue in the form of taxes, fee, and profit for local governments in China [76]. Both the government and private sectors are acquiring agricultural land for infrastructure expansion, the development of economic zones, and real estate. This has serious repercussions for the livelihoods of rural households. More than half of the rural households reported that they suffered livelihood shocks due to land expropriation (Fig 2). The phenomenon of farming land loss and the subsequent formation of landless agricultural communities has given rise to a range of various issues in China. These issues also include concerns surrounding the sustainability of the livelihoods of landless farmers [16,17].

### 4.5. Consequences of land loss on livelihood shocks

The expropriation of land has been found to have negative impacts on the livelihoods of farmers, primarily due to their limited employment opportunities [55]. The logistic regression results showed that there is a negative relationship between the gender of the farmers and livelihood shocks due to land expropriation. Male farmers were 0.61 times less likely to experience livelihood shocks compared to female farmers. There was a negative association between age categories and the land livelihood shocks of the farmers due to land loss. Young farmers were 0.29 times less likely to suffer livelihood shocks due to land expropriation. Low-educated farmers were 2.94 times more likely to suffer livelihood shocks than high-educated farmers due to land expropriation. On the other hand, farmers with a medium level of education were 2.46 times more likely to experience livelihood shocks than farmers with a high level of education. The results depict that farmers with lower education levels were more likely to be affected by land loss as compared to farmers with medium and high education levels. Xu et al. [77] also found that human capital, such as education, is critical to surviving livelihood shocks after land expropriation. Moreover, Zhan [78] and Fitawok et al. [79] who also reported that livelihood shocks are affect by the socio demographic characteristics of the farmers. Tuan [80] also stated that livelihood of the farmers is affected but is conditional on gender, age and education of the land lost farmers.

There is another important result about the purpose of land expropriation’s impact on livelihood shocks. This result revealed that farmers who lost land due to the development of economic zones and infrastructure had lower chances of facing livelihood shocks than those who lost land due to real estate. The reason may be that the development of economic zones and infrastructure is mostly undertaken by the government. The government also offers jobs for land expropriation to the farmers as compensation that may have safeguarded farmers against livelihood shocks. Farmers with complete land loss were 1.70 times more likely to suffer livelihood shocks than those with partial land loss. There was a positive association between distance from home to the city center and livelihood shocks due to land expropriation (Table 4). A 1-kilometer distance from home to nearby city market increases the probability of experiencing livelihood shock by 1.26 times due to expropriation. Xie [81] also reported that the increase in distance from home to nearby city market increases the likelihood of farmers facing livelihood shocks.

**Table 4:** Implications of land expropriation on livelihood shocks.

Variables	β	Std. error	Odd ratios
Gender (1 = Male)	-0.497**	0.223	0.61
Age (1 = Young)	-1.250*	0.431	0.29
Education level (a)			
Low	1.081**	0.452	2.94
Medium	0.903*	0.341	2.46
Marital status (1 = Married)	0.009	0.041	1.01
Family size (1 = Small)	-0.345	0.432	0.71
Land expropriation purpose (b)			
Development of economic zones	-0.413*	0.120	0.66
Infrastructure expansion	-0.392*	0.135	0.67
Land expropriation compensation (1 = Monetary)	1.543	0.612	4.67
Change in household income (1 = Decreased)	0.673	0.544	1.96
Expropriated land (1 = Fully)	0.532**	0.245	1.70
Distance to city market (Kilometre)	0.234**	0.111	1.26
LR chi2	107.231		
Prob. > chi2	0.000		
Log likelihood	678.965		

(a) = base category high education level, (b) = base category real estate

### 4.6. Implications of farming land loss on well-being of farming community

Land expropriation, as a crucial aspect of the urbanization process and it has the potential to have both beneficial and negative repercussions for farmers’ well-being. However, because the land expropriation compensation in monetary terms for confiscated land is typically low, it is debatable whether the remuneration can improve the well-being of landowners [82]. The logistic regression results indicated that the well-being of all farmers was not affected equally, and some farmers’ well-being was affected more than others due to various socioeconomic backgrounds (Table 5).

**Table 5:** Implications of land expropriation on subjective well-being.

Variables	β	Std. error	Odd ratios
Gender (1 = Male)	-0.236	0.332	0.79
Age (1 = Young)	-0.743**	0.331	0.47
Education level (a)			
Low	1.320*	0.216	3.74
Medium	0.700**	0.341	2.01
Marital status (1 = Married)	-0.196	0.181	0.82
Family size (1 = Small)	-0.132**	0.06	0.88
Land expropriation purpose (b)			
Development of economic zone	0.198***	0.100	1.21
Infrastructure expansion	0.167*	0.005	1.18
Land expropriation compensation (1 = Monetary)	0.443**	0.212	1.56
Change in household income (1 = Decreased)	0.255**	0.123	1.29
Expropriated land (1 = Fully)	0.132	0.45	1.14
Distance to city market (Km)	0.431	0.354	1.53
LR chi2	206.143		
Prob. > chi2	0.000		
Log likelihood	745.200		

(a) = base category high education level, (b) = base category real estate.

The econometric model results showed that the subjective well-being of young farmers was 0.47 times more likely to be affected due to land expropriation than that of old farmers. The subjective well-being of rural households with low and medium education levels was likely to be affected more than that of rural households with high education levels. The subjective well-being of the agriculturalists with low education levels was 3.74 times more likely to be affected than that of agriculturalists with high education levels. The subjective well-being of the agriculturalists with medium schooling levels was 2.01 times more likely to be affected than that of agriculturalists with high schooling levels. Zhao et al. [83] also reported in their study that the well-being of farmers is affected differently due to their heterogeneous socioeconomic backgrounds. Tong et al. [84] also a significant relationship between age, education, gender, and well-being of landless farmers.

According to Zhang and Tong [85], a significant number of farmers without land experienced challenges in adapting to the urban setting, primarily due to their limited identity recognition, ultimately affecting their well-being. There was an inverse association between family size and the subjective well-being implications of the farmer’s land expropriation. The subjective well-being of farmers with small family sizes was likely to be affected less by land loss than that of farmers with large family sizes. The results revealed that the subjective well-being of the farmers who lost land due to the development of economic zones and infrastructure was less affected than that of those who lost land due to real estate. Similarly, the subjective well-being of the farmers receiving monetary compensation for expropriation was 1.56 times more likely to be affected after land loss as compared to those who received non-monetary compensation. The subjective well-being of farmers whose income decreased after land expropriation was 1.14 times more likely to be affected than that of farmers whose income did not decrease after land expropriation. Walters et al. [86] also found a negative effect of land expropriation in their study. The previous findings regarding the consequences of farming land loss on well-being present contradictory results. Qin et al. [87] found a negative effect of land loss on rural households’ well-being, while Wang et al. [88] reported a beneficial impact of land loss on rural households’ well-being.

## 5. Conclusion

The phenomenon of converting farmland into non-agricultural uses is attributed to the processes of economic advancement and urban expansion. The conversion of land use significantly alters the traditional way of life of farmers who have been engaged in farming activities. Over the course of the twenty to thirty years, China has achieved remarkable economic development and urbanization. However, this process has resulted in the inevitable expropriation of a significant amount of farmland. The loss of land has a significant impact on the livelihoods, food security, and well-being of farmers. This research explores the impact of land loss on livelihood shocks, food security, and the well-being of farmers. The study is based on survey data collected and employs theoretical and empirical analyses to investigate the effect of land loss on livelihood shocks, food security, and the well-being of farmers. The primary findings of this study are outlined below.

The descriptive findings showed that land expropriation has detrimental effects on the livelihood, food security, and well-being of the farmers. Furthermore, the aforementioned impacts are more harmful among land-expropriated households with a lower educational level, a large family size, and women farmers in less developed rural communities. The results revealed that 27% of households were food insecure before land expropriation, which increased to 39% after land expropriation. Moreover, more than half of the rural households reported that they suffered livelihood shocks due to land expropriation.

The econometric results evinced that gender, age, education level, marital status, family size, and negative changes in income all significantly affect the impact of land expropriation on the food security of rural households. The logistic regression was also used to determine the factors affecting the impacts of land expropriation on livelihood shocks. The findings revealed that farmers with lower education levels were more likely to be affected by land loss as compared to farmers with medium and high education levels. Farmers with complete land loss were 1.70 times more likely to suffer livelihood shocks than those with partial land loss. Moreover, a 1-kilometre distance from home to nearby city market raises the probability of experiencing livelihood shock by 1.26 times due to expropriation. Similarly, the model results regarding the factor determining the impact of land acquisition on the subjective well-being of the farmers indicated that the well-being of all farmers was not affected equally, and some farmers’ well-being was affected more than others due to various socioeconomic backgrounds. For example, the subjective well-being of young farmers was 0.47 times more likely to be affected due to land expropriation than that of old farmers. Similarly, the well-being of the agriculturalists with low schooling levels was 3.74 times more likely to be affected than that of agriculturalists with high schooling levels. The subjective well-being of the agriculturalists with medium schooling levels was 2.01 times more likely to be affected than that of agriculturalists with high education levels.

The implementation of community-based sustainable agricultural programs has the potential to significantly improve food security, livelihoods, and overall well-being for individuals residing in rural areas without access to land ownership. Promoting the establishment of cooperative agricultural collectives or community gardens provides an opportunity for people who lack personal land ownership to engage in collaborative cultivation. In addition, the implementation of focused microfinance programs and skill development efforts in rural areas is of utmost importance for enhancing the overall welfare of individuals without land ownership.

The present research endeavors to enhance comprehension of the consequences of farming land loss on food security, livelihood shocks, and the well-being of the agricultural populace in China. We advocate for the implementation of public policies that provide support to farmers who have been marginalized due to land acquisition. Furthermore, it is imperative that the advancement of land expropriation align with the economic growth of the region.

This study makes a considerable contribution to the literature, but it is not without its limitations. First, sampling bias arising from the exclusion of certain groups may cause the sample to misrepresent the population. Additionally, biases, such as response bias and social desirability bias, can be introduced into data collection techniques, leading participants to give responses that they think are more socially acceptable than those that really reflect their experiences. Third, the complete capture of the multifaceted factors that drive agricultural land acquisitions, including socioeconomic variables and policy shifts, can provide a significant challenge, potentially resulting in oversimplifications in the analytical process. Finally, the accuracy of respondents’ self-reporting is a crucial factor that influences the quality of the data, since it introduces the potential for recall bias or misinterpretation of survey questions. Future studies could explore the relationship between land acquisition and farming welfare by collecting longitudinal data using different sampling techniques in other developing countries.

## Supporting information

S1 Dataset (XLSX)
